# A Study on Fast and Low-Complexity Algorithms for Versatile Video Coding

**DOI:** 10.3390/s22228990

**Published:** 2022-11-20

**Authors:** Kiho Choi

**Affiliations:** School of Computing, Gachon University, Seongnam 13120, Republic of Korea; aikiho@gachon.ac.kr

**Keywords:** versatile video coding, fast VVC, low-complexity VVC

## Abstract

Versatile Video Coding (VVC)/H.266, completed in 2020, provides half the bitrate of the previous video coding standard (i.e., High-Efficiency Video Coding (HEVC)/H.265) while maintaining the same visual quality. The primary goal of VVC/H.266 is to achieve a compression capability that is noticeably better than that of HEVC/H.265, as well as the functionality to support a variety of applications with a single profile. Although VVC/H.266 has improved its coding performance by incorporating new advanced technologies with flexible partitioning, the increased encoding complexity has become a challenging issue in practical market usage. To address the complexity issue of VVC/H.266, significant efforts have been expended to develop practical methods for reducing the encoding and decoding processes of VVC/H.266. In this study, we provide an overview of the VVC/H.266 standard, and compared with previous video coding standards, examine a key challenge to VVC/H.266 coding. Furthermore, we survey and present recent technical advances in fast and low-complexity VVC/H.266, focusing on key technical areas.

## 1. Introduction

Many qualified media services have been provided owing to the advancements in multimedia technology, including content generation, data compression, large-data delivery, rendering technology, and real-time encoding/decoding technology. Based on such technologies, territory broadcasting, movies, on-demand videos, video-based conference calls, video-based mobile communications, video surveillance, real-time remote control, 3D videos, augmented reality videos, and virtual reality video services have generated significant interest among consumers [1].

The popularity of media services is causing network bandwidth problems due to the delivery of various types of media that require a large amount of data for qualified services. A recent Cisco report claimed that video data account for roughly 80% of all Internet data traffic [2]. This aspect is becoming increasingly entrenched because of the recent increase in non-face-to-face activities. Following the COVID-19 pandemic, for example, many activities have been replaced with non-face-to-face multimedia methods, and such video-based solutions are increasing the amount of video data traffic. With the growth in video traffic, video coding techniques capable of reducing video traffic have become more important.

Historically, video coding has been essential to the growth of the media industry. Without the use of data compression technology in the development of digital media, a digital media transformation would be impossible. MPEG-1 [3] was used on CDs, DVDs, and other storage media and played a significant role in changing the use of storage media. MPEG-2/H.262 [4] technology enabled digital broadcasting to replace analog broadcasting. MPEG-4 Advance Video Coding (AVC)/H.264 [5] is a bitstream that has been used as a core transmission format in the Internet era, accounting for more than 80% of all Internet streams worldwide [6]. High-Efficiency Video Coding (HEVC)/H.265 [7] is an ultra-high-definition (UHD) video format used in 4K terrestrial broadcasting, where it plays a leading role [8].

The aforementioned video coding technologies are typically a result of collaboration between two well-known video standard organizations, the ITU-T Video Coding Experts Group (VCEG) and the ISO/IEC Moving Picture Experts Group (MPEG), used to meet the industry needs and consumer demands for a higher resolution, higher quality, and higher frame rate within the context of constrained intra-bandwidth structures. MPEG-2/H.262 was the first video coding standard jointly created by the VCEG and MPEG groups [9]. Its goal was to enable digital television services and has been successful in this endeavor. The VCEG and MPEG groups jointly developed the AVC/H.264 video coding standard, which has been rapidly adopted in online services [10]. Since 2010, the VCEG and MPEG groups formed the Joint Collaborative Team on Video Coding (JCT-VC), which has designed the HEVC/H.265 standard for UHD services [11].

**Figure 1:**
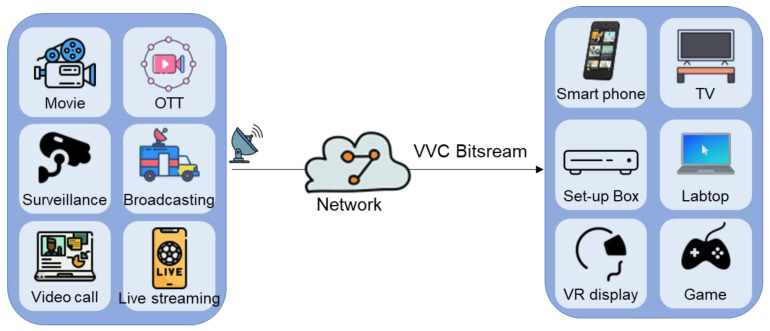
Use case of VVC standard.

Following the completion of HEVC/H.265, there have been calls for a new video coding standard enabling the support of realistic media requiring large numbers of data, such as 8K or higher resolutions, 360-degree virtual reality, screen contents, high dynamic range, a wide chromatic gamut, and adaptive streaming [12], as shown in Figure 1. To properly support realistic media, the VCEG and MPEG groups established the Joint Video Exploration Team (JVET) and began researching important technologies for coding efficiency in 2015. After the study period, Versatile Video Coding (VVC)/H.266 [13] was launched in 2018, the primary objective of which was to achieve a compression capability that is noticeably better than that of HEVC/H.265 and to have the functionality to support a variety of applications with a single profile. The formal standardization period ran from 2018 to 2020, and in October 2020, JVET released the definition of VVC/H.266 version 1.

VVC/H.266 effectively accomplished its goal by utilizing a variety of cutting-edge coding techniques for intra- and inter-prediction, transform, quantization, in-loop filtering, and entropy coding, together with flexible block partitioning. For similar objective and subjective video qualities, VVC/H.266 showed a considerably larger bitrate reduction than HEVC/H.265 [13]. However, the encoding complexity is significantly higher than that of HEVC/H.265 because VVC/H.266 uses many coding tools with flexible partitioning, and the decision of tool on/off and the proper shape of the coding block results in numerous encoding processes. Real-time software and hardware applications may experience a great deal of strain as a result of a high encoding complexity.

To address the issue of the VVC/H.266 coding complexity, numerous studies on fast coding methods with low complexity have recently been conducted. In this study, we aim to provide a comprehensive review of the latest fast and low-complexity encoding methods for versatile video coding. The main contributions of this paper are as follows. (1) We conduct a brief review of the advancements of the VVC/H.266 standard over previous video coding standards. (2) We analyze and identify key challenges in video encoding based on VVC/H.266 coding. Finally, (3) we conduct a comprehensive survey of recent advances in fast and low-complexity video coding methods, which are classified into specific coding areas for low complexity.

The remainder of this paper is organized as follows. Section 2 provides a detailed overview of the VVC/H.266 standard and some key technologies. In Section 2, the VVC/H.266 coding performance and complexity are analyzed, and challenging issues are presented. In Section 3, recent studies on the fast and low-complexity VVC/H.266 are reviewed from key technical perspectives. Finally, Section 4 provides some concluding remarks.

## 2. Overview and Complexity Analysis of VVC/H.266 Standard

### 2.1. VVC/H.266 Standard

The VVC/H.266 standard uses a block-based hybrid coding framework, which is similar to earlier video coding standards. To improve the coding performance, the VVC/H.266 standard employs novel coding tools with a flexible block partitioning method. As with all previous video coding standards, the VVC/H.266 standard employs intra-prediction to reduce the spatial redundancy, inter-prediction to reduce the temporal redundancy, transform coding of the residuals to further reduce the spatial statistical redundancy, and in-loop filters to enhance the quality of the reconstructed video. The VVC/H.266 standard specifically employs a multi-type tree (MTT) structure as quad-tree, binary, and/or tri-tree block partitions using a 1:2 to 1:8 ratio between the width and height of the block [14,15]. In contrast to the HEVC/H.265 block partitioning method, there are no prediction units for prediction or the transformation sizes, nor are there any transform units for a transformation. In general, intra- and inter-predictions are used in block or sub-block units, followed by transformations, quantization, and entropy processes for residual coding and in-loop filter chains to improve the visual quality.

Intra-prediction is improved by increasing the number of directional angles from 35 to 93 to provide an accurate prediction using the new VVC/H.266 partitioning shapes [16]. To increase the number of original pixels in the prediction, new intra-tools such as a position-dependent prediction combination [17], multiple reference line (MRL) [18], matrix-based intra-prediction [19], cross-component linear model [20], and intra-sub-partition (ISP) [21] have been adopted. Eventually, by improving the existing prediction tools and adopting new intra-tools, the intra-coding performance has improved significantly.

To enhance the coding performance by minimizing the temporal duplication between sequential frames, many inter-prediction tools have been adopted in the VVC/H.266 standard. According to whether motion data are shared across an entire block, newly adopted inter-prediction tools in VVC/H.266 can be typically divided into two groups. For instance, all block-based inter-prediction tools include history-based motion vector prediction [22], merge with motion vector difference [23], symmetric motion vector difference [24], adaptive motion vector resolution [25], geometric partitioning mode (GPM) [26], bi-prediction with CU-level weights [27], and combined intra- and inter-prediction [28]. By contrast, sub-block-based methods include affine motion [29], sub-block-based temporal motion vector prediction [30], decoder side motion vector refinement [31], bidirectional optical flow [32], and prediction refinement with an optical flow [33]. Owing to the introduction of these cutting-edge techniques, VVC/H.266 inter-prediction has been significantly improved compared to HEVC/H.265 inter-prediction.

A multiple transform selection (MTS) [34], sub-block transform [35], and non-separable secondary transform [36] have been used toward a transform and quantization of the residual coding for both inter- and intra-compressed blocks. The quantization in VVC/H.266 adopts dependent quantization, which can be a form of sliding block vector quantization [37]. Luma mapping with chroma scaling, a cross-component adaptive loop filter, and an adaptive loop filter are all new in-loop filtering filters introduced in VVC/H.266 [38].

The VVC/H.266 standard improved all coding components of the hybrid coding structure and incorporated advanced coding tools to achieve the challenge goal of improving the coding efficiency by 50% over the prior coding standard (i.e., the HEVC/H.265 standard). Although these advancements have contributed to achieving the objectives of VVC/H.266, an increase in complexity was unavoidable, which needed to be resolved.

### 2.2. Complexity Analysis

During the official standard phase, JVET maintained a VVC/H.266 test model called VTM [39], which served as reference software for VVC/H.266 testing. The primary goal of VTM is to provide an exemplary reference implementation of a testbed VVC/H.266 encoder and decoder and to evaluate the proposed tools. In addition, JVET created the common test condition (CTC) [40] to evaluate the coding performance under the same testing environment. CTC specifies the test conditions based on the following scenarios that are commonly used in the real world: (1) all intra (AI), in which all frames are encoded with I slices; (2) random access (RA), which uses picture reordering with a random access picture every 1 s; (3) low delay with B slices (LB), in which frame reordering is not allowed and only the first frame is encoded using an I slice followed by B slices; and (4) low delay with P slices (LP), in which frame reordering is not allowed and only the first frame is encoded using I slices followed by P slices. Based on the four CTC scenarios, the coding performance of VVC/H.266 can be compared with the previous coding standard.

**Figure 2:**
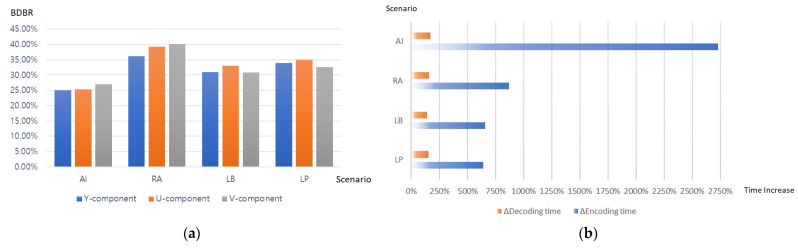
VVC/H.266 performance compared to HEVC/H.265. (**a**) VVC/H.266 coding performance over HEVC/H.265, and (**b**) VVC/H.266 complexity compared to HEVC/H.265.

Figure 2 summarizes the performance of VTM10.0 [39] over HM16.22 [41] for all configurations in a JVET CTC environment [42]. Bjøntegaard delta bitrates (BDBR) [43] are used to compare the coding performance of VTM10.0 as a whole package to HM16.22. The runtime was used as an estimation of the codec complexity to check the encoding and decoding complexity, with the following *T* measurement: *T* = *Ttest*/*Tanchor* * 100%, where *T_test_* and *T_anchor_* represent the runtimes of the tested and anchor methods, respectively. A value of 100% indicates that there is no difference in runtime.

According to Figure 2, VTM10.0 reduces the luma BDBR by 25.1%, 36.1%, 30.9%, and 33.9% compared to HM16.22. In the AI, RA, LB, and LP configurations, the encoder runtime of VTM10.0 is approximately 27, 9, 7, and 6 times slower than that of HM16.22. In all configurations, the decoder runtime of VTM10.0 is approximately 1.5 times that of HM16.22. The increase in the encoding and decoding times is primarily due to the addition of new tools that require additional rate-distortion checks for selecting the best mode for a block, and the flexible block partitioning also necessitates a more exhaustive search for finding the optimal partitioning for a coding tree unit (CTU). For instance, the encoder must compute the bit rate and distortion of all feasible combinations of the block partitions and compressible coding tools before deciding on the optimum partition and tool for a given block. Such computations used for selecting the best partition with the best coding tools result in a significant increase in the VVC/H.266 complexity.

## 3. Fast and Low-Complexity Coding for VVC/H.266

**Figure 3:**
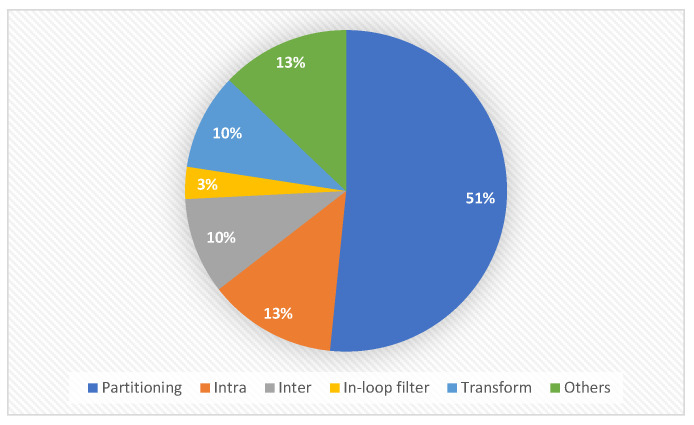
Classification of research into fast VVC.

Several recent efforts have been made to address the complexity of VVC/H.266. The majority of studies on fast and low-complexity coding of VVC/H.266 have focused on reducing the complexity of the block partitioning. Based on our search of recent papers on this topic, we identified that more than half are related to the fast method for block partitioning, as shown in Figure 3. Considering that block partitioning involves all encoding processes associated with the coding tools, focusing on fast partitioning makes sense. As a result, in this section, we look at recent advances in fast and low-complexity video coding methods, which are divided into four categories: (1) fast methods for an early split mode decision, (2) fast methods for an early coding unit (CU) depth decision, (3) fast methods for coding tools, and (4) low-complexity platform-dependent methods.

### 3.1. VVC/H.266 Block Partitioning

**Figure 4:**
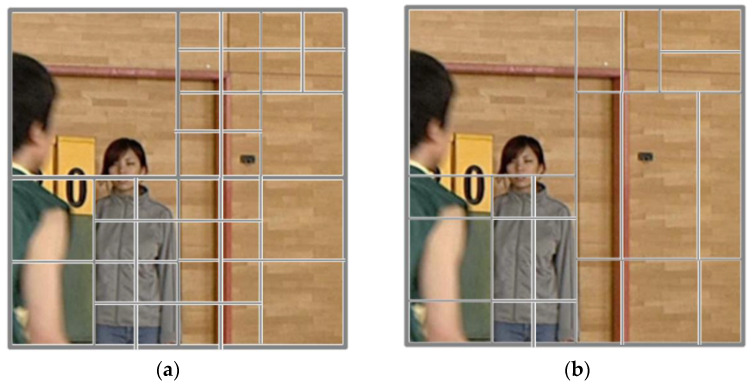
Block partitioning: (**a**) QT in HEVC/H.265 and (**b**) QT/BT/TT in VVC/H.266.

This subsection describes the basic partitioning process used in VVC/H.266 encoding before reviewing the fast and low-complexity methods applied for partitioning. The block partitioning used in VVC/H.266 achieves a significant coding performance by allowing for a flexible block size. Such flexible partitioning can create adaptive CU partitions based on the video image characteristics using the newly introduced MTT. Figure 4 shows an example of VVC/H.266 block partitioning versus HEVC/H.265 block partitioning. As illustrated in the figure, VVC/H.266 can determine a more flexible CU than HEVC/H.265 based on the frame content in the frame. This flexibility is achieved by layering the binary tree (BT) and tri-tree (TT) partitioning on top of quadtree (QT) partitioning. This is one of the major differences between BT and TT partitioning, which can produce nonrectangular CU shapes depending on the content.

**Figure 5:**
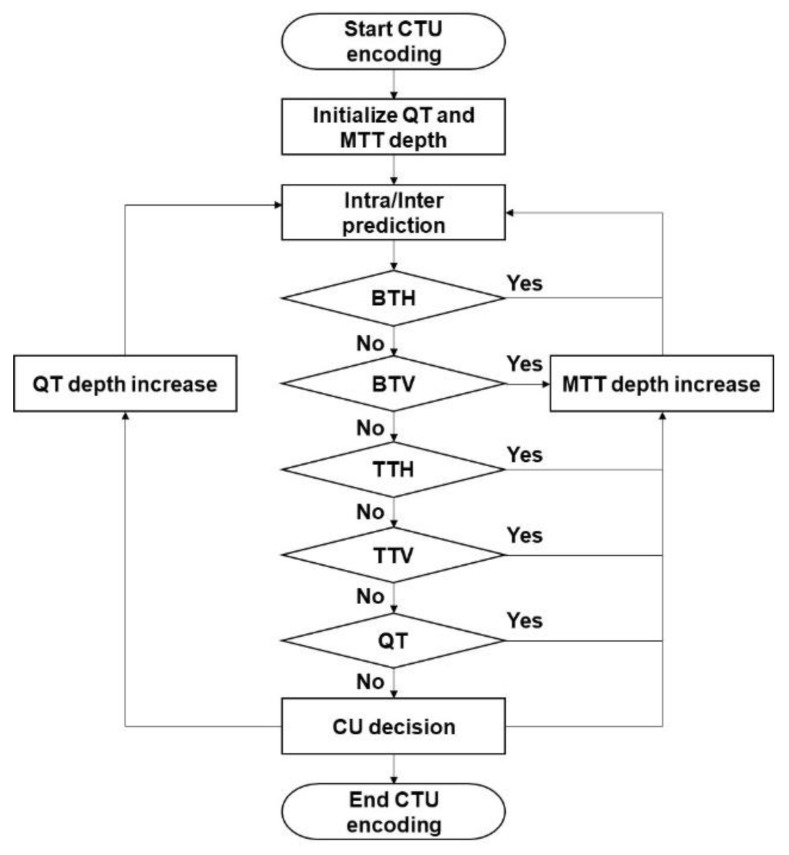
Flowchart of VVC/H.266 block partitioning.

Nonetheless, the introduction of BT and TT significantly increases the encoding time. To determine the best-fitting CU block shape, an exhaustive evaluation of all possible QT, BT, and TT block shapes results in significantly more recursive calls to the coding tool functions than HEVC/H.265 using QT. Figure 5 depicts the encoding process of VVC/H.266 for determining the CU as well as the corresponding split mode of the tree at a given depth. Binary tree splits were first evaluated horizontally and vertically at the given depth, followed by tri-tree splits horizontally and vertically, and the quadtree was then evaluated. When MTT (i.e., binary tree and tri-tree) is applied to the QT leaf, only BT and TT are permitted, whereas QT is prohibited for all subsequent nodes. Each MTT node has the option of being non-split (as an MTT leaf) or divided into two child MTT nodes by a horizontal binary tree (HBT), two child MTT nodes by a vertical binary tree (VBT), three child MTT nodes by a horizontal ternary tree (HTT), or three child MTT nodes by a vertical ternary tree (VTT). The two MTT child nodes in the BT scenario were of the same size, with each being half the size of the parent MTT node. The three MTT child nodes in the TT scenario have a splitting ratio of 1:2:1 and are one-fourth, one-half, and one-quarter the size of the parent MTT node, respectively. Choosing the split mode and depth for optimal partitioning requires a significant amount of encoding time. Two approaches have been investigated to reduce the encoding time of the chosen partitioning.

### 3.2. Fast Method on Early Split Mode Decision

The first approach is to investigate the split mode for an early split mode determination or to skip some of the split modes in the mode evaluation. At a given depth, five split modes were evaluated in the VVC/H.266 partitioning, i.e., HBT, VBT, HTT, VTT, and QT. With this approach, the partitioning process involves a reduced number of evaluations to determine the split mode by skipping unnecessary evaluations at a given depth. This approach saves a significant amount of encoding time by reducing computations in a skipped mode evaluation. The following studies were investigated from this perspective.

Park et al. [44] proposed a simple early decision method based on a probabilistic approach that can effectively reduce the TT complexity by exploiting rate distortion (RD) costs from previously encoded CU data. The authors specifically investigated the split modes determined after the encoding process, followed by an examination of the relationship between the TT split and the texture of the contents, which can be estimated using the RD cost of the partitioning shape. The authors developed a TT decision model based on the Bayesian probability approach, and the proposed method adaptively skips the TT partition evaluation process, thereby saving a significant amount of encoding time with a marginal coding loss.

In [45], Park et al., proposed a fast TT decision method by exploiting the statistical information of coded bitstreams representing the correlation with the TT and developed two useful types of features: intra-prediction information and block information using QT, HBT, and VBT during an evaluation. The authors created a neural network and model trained using these two features. The developed model can efficiently determine whether the TT partitioning process is involved. Consequently, the proposed method efficiently reduces the encoding time related to the TT partitioning in the encoder.

In [46], Zhao et al. proposed a fast CU partitioning method that investigates the just noticeable difference (JND) model and motion state by combining such information with a decision tree to develop a CU partition decision strategy oriented toward the perceived quality of the human visual system (HVS). The authors developed a threshold that determines whether an individual split mode is evaluated at a given depth based on an analysis of the JND model and the motion state, and used the threshold to skip an individual mode evaluation.

In [47], Zhang et al., proposed a fast CU decision method based on the DenseNet network that trains a convolutional neural network (CNN) to predict the probability that the edges of the 4 × 4 blocks in each 64 × 64 block skip the computation of the unnecessary rate distortion optimization (RDO) and accelerates the coding process. The proposed CNN model analyzes the texture of four 64 × 64 blocks of content, and the produced probability of the 4 × 4 blocks in the 64 × 64 is used to determine the RDO evaluation of the individual split modes.

In [48], Saldanha et al., proposed a fast CU partitioning method using a light gradient boosting machine (LGBM) to reduce the VVC/H.266 intra-coding time. The authors trained five LGBM classifiers offline to avoid evaluating the split modes that were likely to be skipped. The generated LGBM classifiers are trained using features extracted from the texture, coding, and coding context information, and the classifiers determine whether each split mode is applied at a given depth. The authors emphasized that the proposed LGBM classifiers can handle a wide range of video characteristics and resolutions, allowing them to support many applications while requiring a relatively short encoding time.

Table 1 summarizes the fast methods for the early split-mode decision. As shown in the table, the proposed methods can reduce the encoding time by 34% to 54% with a relatively marginal coding loss by estimating the promising split mode at the early stage rather than evaluating all possible split modes.

### 3.3. Fast Method Applied to Early CU Depth Decision

The second approach is to determine the portioning depth at an early stage. In the VVC/H.266 partitioning process, all possible combinations of CU shapes are evaluated under the allowed depths of QT, BT, and TT. According to Figure 5, QT, HBT, VBT, HTT, and VTT are evaluated sequentially, followed by the same portion mode at the next depth with a reduced block size depending on the parent CU block. Such a recursive calling process for evaluating all possible block combinations consumes a considerable amount of the encoding time. If the encoder determines the optimal CU blocks at the early stage, it significantly reduces the useless evaluation process for further depth of partitioning. From this viewpoint, the following studies were conducted to develop a fast-encoding method for determining the CU depth at an early stage.

In [49], Zhang et al., proposed a fast-partitioning method for early depth and intra-mode decisions, and investigated a fast CU partitioning based on a random forest classifier (RFC) model and fast intra-prediction mode decision using the texture region features. First, the proposed method classifies the texture complexity of the current CU using information extracted from the mean of the absolute difference between pixels to define the difference between each pixel and its surrounding pixels for fast CU partitioning. The extracted feature is then used in the RFC to determine the coding depth by providing a split threshold. Furthermore, the authors exploited the correlation with the pixel similarity in the corresponding direction, and then proposed a fast intra-mode decision using the energy of the CU in four directions based on the texture information using the Canny operator to avoid an unnecessary intra-prediction mode evaluation.

In [50], Zhang et al., proposed a split-mode method and a fast CU depth decision approach. First, the proposed method determines whether a CU is divided by calculating the texture complexity using the rough mode decision (RMD)-based cost (JRMD) and angular second moment (ASM). The decision is made by comparing the ASM value of the current CU with the derived threshold. Second, the authors presented a fast split-mode decision method that uses a threshold generated from the SAD of each direction to determine which split modes are skipped for the current depth.

Zhang et al. [51] proposed a fast Bayes-based CU partitioning method by leveraging the relationship between JRMD and split depth determination. The proposed method determines whether a further split is required based on the threshold generated by the JRMD. Furthermore, the authors use a deblocking filter (DBF) to check the texture information of the current block and embed such information to determine which split modes can be discarded in the evaluation.

Fan et al. [52] proposed a fast CU partitioning method to determine whether to split a CU by exploiting the texture smoothness using the variance of the given block and a Sobel operation for terminating further partitions. To this end, the authors derived a threshold using the Sobel operation by comparing the variance of the block. Furthermore, the authors presented a gradient of the texture of the contents to only select one partition from the five split modes. The authors generated two additional thresholds to determine which split modes were evaluated for the proposed method.

In [53], Yang et al., proposed a fast CU partitioning method and a fast intra-mode decision method. First, the authors analyzed the statistics between the determined CU size and the texture information, and then proposed a statistical learning-based fast depth decision method derived by calculating the features to measure the texture characteristics and context correlations. These features are then used as input values to the classifiers to determine whether CU processing is required at higher depths. In addition, the authors exploited the Hadamard cost of each directional mode in the MPMs, followed by a gradient descent-based search to find the optimal intra-mode prediction. The proposed intra-prediction methods reduce the RDO computations of unnecessary intra-modes.

In [54], Tang et al., proposed a fast-partitioning method for intra- and inter-coding. The block-level-based Canny edge detector is used for intra-coding to extract the edge features to conduct an early termination of the split depth. Similarly, for inter-coding, the authors exploited the temporal correlation of consecutive frames in the video to generate a threshold based on the difference between three consecutive frames to determine whether a future split is required. Additionally, the authors presented a method for a fast split-mode decision using thresholds and a Canny edge detector skipping vertical or horizontal partition modes.

Liu et al. [55] proposed a fast-partitioning method for inter-frame coding based on spatiotemporal information by utilizing the motion features and texture complexity of the current coding block. The authors calculated the average sum of the square difference (ASSD) of the luma values of the current block and the reference block at the same position in the co-located reference frame and its derived threshold to determine whether further CU partitioning is required. Furthermore, the authors used the derived threshold to bypass the individual split mode during a split mode evaluation.

In [56], Li et al., proposed a deep MSE-CNN model that combines a conditional convolution and sub-networks with the adequate network capacity to determine the CU partition at an early stage of the CU partitioning, which can skip unnecessary evaluation processes on unused CUs. MSE-CNN with a 128 × 128 CTU input is used in the proposed method to extract a collection of 128 × 128 feature maps. The five split modes are applied sequentially using the feature maps, which are then sent into a sub-network to anticipate one of the CU split modes. The network then decides whether further splitting is necessary.

In [57], Chen et al., proposed a fast approach for VVC/H.266 intra-coding by utilizing the human visual system and a machine learning technique. To identify the visually distinguishable pixels in a CU, the author used a perceptual model of the human eyes, which shows only perceptible differences. The quantization parameter (QP) and the horizontal and vertical projections of visually distinct pixels are used as the input values of the random forest machine learning models to predict the CU partitions and remove computations regarding unnecessary split decisions.

In [58], Yea et al., proposed a CNN-based fast split mode decision method for inter-coding by utilizing the original and residual image of a CU, the picture order count, and the CU-level QP value. The proposed multi-level tree CNN method predicts which of the five split modes will be evaluated to reduce the time complexity of the inter-picture prediction mode evaluation.

Pan et al. [59] proposed an MF-CNN-based CU partitioning early termination method to streamline the CU partitioning process by utilizing texture and motion activity features for a fast inter-coding approach. The authors trained a CNN model to determine the CU depth using the luma component, residuals, and bidirectional motion field of the CU. In addition, the authors presented an early decision method for the merge mode. According to a statistical analysis, the authors discovered that determining the merge mode early can save a significant amount of encoding time. Similar to early CU partitioning, the authors applied an MF-CNN to determine whether the merge mode was the best inter-coding mode.

The method in this section uses texture or motion information to determine the early stage of depth during the CU encoding processing. Based on the observation that such information is strongly related to the determined partitions, the proposed methods apply novel algorithms developed to determine the optimal CU size and partitions at an early stage. As shown in Table 2, the fast methods for early CU depth decisions can save 12% to 58% of the total encoding time by skipping unnecessary computations when evaluating the combination of the tool and CU size.

### 3.4. Fast Method for Coding Tools

Many tools have been adopted in VVC/H.266 to improve the coding performance for both intra- and inter-coding. Among the newly adopted and updated tools, several computations are required to determine the best modes or motion information. In this section, we look at studies that have been conducted to reduce the number of computations required by individual coding tools. Most studies reduce the number of RDO checks by reducing the modes or determining the best mode early in the process.

Dong et al. [60] proposed a fast intra-mode decision method based on two efficient algorithms. First, the proposed method efficiently eliminates any unnecessary RDO processing by skipping the intra-block copy (IBC) and ISP tools from learning-based classifiers and excluding subsequent candidates in a complete mode list. The authors took advantage of the early depth decision to improve the speed by categorizing CUs into three groups using texture and coding information.

In [61], Tun et al., proposed a fast intra-prediction mode selection for intra-prediction mode RDO computations. The authors first examined the relationship between the RD costs of the RDO processes and SATD costs of the RMD processes and then developed a threshold that can be used to determine which intra mode will be promising. Based on the threshold, the proposed method can include only a small number of intra-predictions in the RDO evaluation process, which is time consuming.

In [62], Park et al., focused on the complexity reduction of ISP. ISP is newly adopted in VVC/H.266, which provides a flexible block shape for an intra-prediction. By referring to closely neighboring reconstructed pixels, such a flexible block shape can provide a more accurate prediction. However, ISP is one of the more time-consuming tools in VVC/H.266 intra-coding. In this paper, the authors proposed a method for skipping an ISP mode that requires an RD-based search to save the encoding time associated with the ISP coding tool by utilizing the relation between ISP and the MRL.

Tsang et al. [63] proposed a fast prediction network based on deep learning for screen content coding (SCC) tools. To handle graphically generated or mixed layered contents, SCC coding tools in VVC/H.266, such as IBC and Palette (PLT), are included in version 1. The characteristics of the contents differ from those of normal natural contents, and such SCC tools are commonly used for SCC contents. The authors created a CNN-based model that efficiently distinguishes between SCC and natural content. The proposed method can efficiently apply the encoding path associated with the content characteristics by applying the CNN model and classifying all 4 × 4 sub-blocks within each 64 × 64 CU, regardless of whether they are a natural content block or screen content block.

Park et al. [64] proposed a fast-encoding method to facilitate an affine motion estimation (AME) process by utilizing features that reflect the statistical characteristics of the CU partition and AME. AME is a newly adopted tool in VVC/H.266, which significantly improves the exit coding performance for non-translational motions in the content. One issue with AME is that it requires numerous computations to achieve a more accurate motion prediction. The authors proposed a method that uses statistical features to skip redundant AME processes by utilizing the determined inter-mode and RD costs available in the conventional motion estimation process.

In [65], Zhang et al., proposed a fast geometric prediction merge mode decision algorithm for VVC/H.266 based on the CU gradient by comparing the mean value of the gradient in four directions to determine whether the GPM can be terminated early. The GPM is a newly adopted tool in VVC/H.266 that can handle moving objects that are not rectangular in shape. The GPM also necessitates numerous computations to find the best combination of two triangular shapes in the inter-coding. The authors developed a method that uses the Sobel operator template and checked the promising directions for use of the GPM, thereby determining the promising object texture for GPM usage.

In [66], Guan et al., proposed a fast AME method based on spatial texture features and the time correlation by calculating the texture and Prewitt gradient and exploiting the best prediction mode of the current block. The enormous computational complexity of AME has motivated the development of the method proposed in this paper, as described in [1]. The proposed method calculates the texture complexity by using the histogram of the block and the texture boundary with the Prewitt operator. The generated texture complexity was used to terminate the AME early, and the accumulated information between the parent and child CUs was used to skip the inter-prediction mode evaluation.

In [67], Fu et al., proposed a fast two-stage method that uses spatial coding statistics and primary transform information to terminate the RDO process of MTS by exploiting the correlations between the RD cost of the primary transform and the RD costs of the child CUs. MTS is a newly adopted tool in VVC/H.266 that, in addition to DCT2, supports the DST7 and DCT8 kernels. By checking four additional transform modes in the core transform, MTS significantly improves the coding performance while consuming a large amount of computational complexity. To reduce the number of MTS computations, the authors presented a procedure that efficiently skips the RDO computations of the MTS mode evaluation if the sum of the RDO costs of the child CUs is greater than the sum of the RDO costs of the parent. In addition, the method employs early termination of the MTS.

In [68], Choi et al., proposed a low-complexity intra-coding scheme applying the downsampling- and upsampling-based fast method by changing the original video size for encoding/decoding and recovering the quality of the reconstructed video using the CNN-based super-resolution network. Additionally, the authors investigated the intra-coding tools in VVC/H.266 to further reduce the encoding complexity by introducing an optimal tool combination under the proposed scheme, the results of which showed significant coding savings with little coding gain.

Table 3 summarizes fast tool-based methods. Such methods can reduce the total encoding time by 4–69%, as shown in the table. Because each tool contributes a different amount to the overall encoding time, the reduction in the encoding time varies depending on the tools used. For example, the AI scenario consumes a significant amount of encoding time for intra-mode selection, and thus the reduction in complexity in intra-mode has a greater impact than other tools. However, the reduction in [62] was relatively small because ISP accounted for only a minor fraction of the total encoding time. The results of [68] are an interesting aspect of the table. Under an AI scenario, the advanced method using upsampling/downsampling with the tool selection demonstrated a relatively high reduction in the encoding time, and even a coding gain of approximately 4.6%.

### 3.5. Platform Dependent Low-Complexity Methods

Efforts have been made to develop a low-complexity method for VVC/H.266 implementation. The transform module was primarily targeted for hardware implementation. The transform module is the most complex module used in a decoder implementation. In particular, in VVC/H.266, the use of MTS causes the decoder to become more complex by adding DST-VII and DCT-VIII with an increased transform size of up to 64 × 64.

Kammoun et al. [69] proposed a forward inverse 2D hardware implementation of an approximate transform core for VVC/H.266 using low-cost adjustment stages on a DCT-II variant to approximate the DST-VII and DCT-VIII transform types. In this study, the authors also proposed a low-complexity-based hardware implementation of the approximate VVC/H.266 transform process.

Hamidouche et al. [70] proposed a low-complexity-based multiple-transform selection module for VVC/H.266 hardware implementation. The authors exploited the approximation of the DST-VII and DCT-VIII transforms to reduce the hardware complexity and memory requirements and thereby implement the VVC/H.266 MTS, particularly on hardware chips with reduced logic and memory resources.

Additional effort has been made to investigate low-complexity VVC/H.266 implementations in software (SW) implementations. Open-source SW-based codecs are widely used in the industry. Looking at the history of standard codecs, it is clear that the timely availability of SW-based codecs has influenced the success of standard codecs; therefore, VVC/H.266 SW-based codec development is regarded as extremely important.

In [71], Wieckowski et al., introduced VVenC as an open-source VVC/H.266 SW encoder implementation. The authors claimed that the open-source SW codec VVenC with the fastest configuration runs over 140 times faster than VTM while providing at least a 10% bitrate reduction compared to HM.

In [72], Wieckowski presented fast partitioning strategies for VVC/H.266 and their implementation in an open-source optimized SW encoder (e.g., VVenC). The authors proposed the following partitioning strategies: skip condition, skip with sub-split skip, split cost prediction adaptation, test ternary split parallel to a better binary split, and inverse split order in low-depth configurations.

In [73], Brandenburg et al., presented Pareto-optimized coding configurations for VVenC. The authors approximated the Pareto set of construct spaces during the iterative process, including search spaces that extend the encoding tool and search spaces that organize fast algorithms. Using pre-grouping tools and options, the proposed method can adaptively apply coding tools and partitions to targeted complexities and ordered sets.

Table 4 summarizes the platform-dependent low-complexity methods mentioned in this section. As shown in the table, the performance varies depending on the target platform. The number of operations and size of the memory and/or area are the most important measurement factors for hardware implementation. Accordingly, the methods in [69,70] reported performance improvements with the coding loss. The methods in [71,72,73] are aimed at producing an SW codec that can be used directly in the industry. It is critical to provide an encoding/decoding that can be applied in real time. For this purpose, the authors developed an open-source SW codec and presented an increase in speed.

## 4. Conclusions

In this study, we reviewed the VVC/H.266 standard against previous video coding standards and analyzed and identified key challenges in video encoding based on VVC/H.266 coding. Furthermore, we surveyed and presented recent technical advances in fast and low-complexity VVC/H.266, taking key technical areas into account. Although the latest video coding standard, VVC/H.266, achieved an improvement in coding performance of approximately 50% compared to HEVC/H.265 by incorporating new advanced technologies with flexible partitioning, the increased complexity of the encoding must be overcome before it can be made available on the market. To address the issue of VVC/H.266, significant effort has been made in developing methods for reducing the encoding/decoding of VVC/H.266. One of the attempted approaches is investigating the early CU depth and split mode decision methods during the partitioning process, which requires the majority of the VVC/H.266 encoding time. Texture analysis, statistical analysis, syntax correlation, and machine learning-based classification were used to estimate the CU depth and split mode. Some methods concentrate on increasing the speed of individual coding tools, whereas others have used practical hardware and software implementations. In conclusion, studies on fast and low-complexity VVC/H.266 algorithms are important and will lead to a promising direction for the success of this standard in academic and industrial communities.

## Figures and Tables

**Table 1:** Summary of fast methods on early split mode decision.

Paper	Tech Area	Key Feature	Anchor	Scenario	T (%)	BDBR (%)
[44]	Intra partition, Fast split mode decision	Bayesian probability approach, Adaptive TT skipping method	VTM4.0	AI	−34	1.02
[45]	Intra partition, Fast split mode decision	CNN model, Adaptive TT skipping method	VTM4.0	AI	−27	0.44
[46]	Intra partition, Fast split mode decision	JND model, Adaptive split mode skipping method	VTM7.0	AI	−48	0.79
[47]	Intra partition, Fast split mode decision	CNN model, Split mode estimation	VTM10.0	AI	−46	1.86
[48]	Intra partition, Fast split mode decision	CNN model, Split mode estimation	VTM10.0	AI	−54	1.42

**Table 2:** Summary of fast methods on early CU depth decision.

Paper	Tech Area	Key Feature	Anchor	Scenario	T (%)	BDBR (%)
[49]	Intra partition, Fast depth decision, Fast split mode decision	Forest classifier model, Canny operator-based texture analysis	VTM4.0	AI	−54	0.93
[50]	Intra partition, Fast depth decision, Deblocking filter	JRMD and intra-mode analysis, SAD-based texture analysis	VTM7.0	AI	−48.58	0.91
[51]	Intra partition, Fast depth decision, Fast split mode decision	JRMD-based depth analysis, DBF texture information analysis	VTM11.0	AI	−56.08	1.3
[52]	Intra partition, Fast depth decision, Fast split mode decision, Intra-mode selection	SAD and Sobel operator-based texture analysis	VTM7.0	AI	−49.27	1.63
[53]	Intra partition, Inter partition, Fast depth decision, Fast split mode decision	Texture information analysis, Trained model, Gradient descent-based search	VTM2.0	AI	−62	1.93
[54]	Inter partition, Fast depth decision, Fast split mode decision	Canny operator-based texture analysis, Temporal correlation analysis	VTM4.0	AIRA	−36−31	0.711.34
[55]	Intra partition, Fast depth decision	Temporal correlation analysis	VTM11.2	RA	−22	1.34
[56]	Intra partition, Fast depth decision	CNN model, Split mode, and depth estimation	VTM7.0	AI	−46	1.32
[57]	Inter partition, Inter-mode decision, Fast depth decision, Fast split mode decision	Forest classifier model, Human visual system analysis	VTM7.0	AI	−41	1.14
[58]	Inter partition, Inter-mode decision, Fast depth decision, Fast split mode decision	CNN model, Split mode, and depth estimation	VTM11.0	RA	−12	1.01
[59]	Intra partition, Fast depth decision, Fast split mode decision	CNN model, Split mode, and depth estimation	VTM6.0	RA	−31	3.18

**Table 3:** Summary of fast tool-based methods.

Paper	Tech Area	Key Feature	Anchor	Scenario	TS (%)	BDBR (%)
[60]	Intra-prediction, Fast depth decision	Learning-based classifier, Intra-prediction estimation	VTM10.0	AI	−53	0.93
[61]	Intra-mode	SATD-based intra-mode estimation	VTM5.0	AI	−21	0.88
[62]	Intra-prediction, ISP	ISP and MRL analysis	VTM14.0	AI	−4	0.04
[63]	Intra-prediction, IBC, PLT	CNN model, Local block analysis	VTM9.2	AI	−30	2.42
[64]	Inter-prediction, AME	Statistical analysis	VTM3.0	RA	−37	0.1
[65]	Inter-prediction, GPM	Sobel operator-based analysis, Direction analysis	VTM8.0	RA	−14	0.14
[66]	Inter-prediction, AME	Prewitt operator-based analysis, Histogram analysis	VTM11.0	RA	−15.5	0.55
[67]	Transform, MTS	DCT cost analysis	VTM3.0	AI	−23	0.16
[68]	Framework	Down/upsampling, Tool on/off analysis	VTM12.0	AI	−69	−4.6

**Table 4:** Platform-dependent low-complexity methods.

Paper	Tech Area	Key Feature	Anchor	Scenario	Performance	BDBR (%)
[69]	Transform, Hardware implementation	Low-cost DCT-II implementation, Approximate DST-VII, DCT-VIII	VTM3.0	AI	12% of Alms, 22% of registers, and 30% of DSP blocks	0.15
[70]	Transform, Hardware implementation	Low-cost DCT-II implementation, Approximate DST-VII, DCT-VIII	VTM3.0	AIRA	5.37%, 68%, 84%, and 92% of multiplication savings with respect to transform sizes N = 8, 16, 32, and 64	0.090.01
[71]	Software implementation	Five predefined presetting different encoding speed/compression quality offsets	VTM12.0	RA	30 × faster	12
[72]	Software implementation, Partition	Split mode and depth estimation	VTM12.0	RA	42% speedup of encoding	1.3
[73]	Software implementation, Tool combination	Pareto set, Pre-grouping tools and options	HM16.22	RA	25% speedup of encoding	−38

## Data Availability

Not applicable.

## Abbreviations

List of abbreviations used in this work.

**Acronym**	**Description**	**Acronym**	**Description**
AI	All intra	LB	Low delay with B-slices
AME	Affine motion estimation	LGBM	Light gradient boosting machine
ASM	Angular second moment	LP	Low delay with P-slices
ASSD	Average sum of the square difference	MPEG	Moving picture experts group
AVC	Advance video coding	MRL	Multiple reference line
BDBR	Bjøntegaard delta bitrates	MTS	Multiple transform selection
BT	Binary tree	MTT	Multi-type tree
CNN	Convolutional neural network	PLT	Palette mode
CTC	Common test condition	QP	Quantization parameter
CTU	Coding tree unit	QT	Quadtree
CU	Coding unit	RA	Random access
DBF	Deblocking filter	RD	Rate distortion
GPM	Geometric partitioning mode	RDO	Rate distortion optimization
HBT	Horizontal binary tree	RFC	Random forest classifier
HEVC	High-efficiency video coding	RMD	Rough mode decision
HTT	Horizontal ternary tree	SCC	Screen content coding
HVS	Human visual system	SW	Software
IBC	Intra-block copy	TT	Tri-tree
ISP	Intra sub-partition	UHD	Ultra-high-definition
JCT-VC	Joint collaborative team on video coding	VBT	Vertical binary tree
JND	Just noticeable difference	VCEG	Video coding experts group
JRMD	Rough mode decision-based cost	VTT	Vertical ternary tree
JVET	Joint video exploration team	VVC	Versatile video coding
